# Dissecting the Effects of Simulated Cattle Activity on Floristic Composition and Functional Traits in Mediterranean Grasslands

**DOI:** 10.1371/journal.pone.0079822

**Published:** 2013-11-20

**Authors:** Iker Dobarro, Carlos Pérez Carmona, Begoña Peco

**Affiliations:** Terrestrial Ecology Group (TEG), Department of Ecology, Universidad Autónoma de Madrid, Madrid, Spain; Institute of Botany, Czech Academy of Sciences, Czech Republic

## Abstract

Livestock exerts direct and indirect effects on plant communities, changing colonization and extinction rates of species and the surrounding environmental conditions. There is scarce knowledge on how and to what extent these effects control the floristic and functional composition of plant communities in grasslands. We performed an experiment that included several treatments simulating trampling, defoliation, faeces addition and their combinations in a Mediterranean scrub community grazing-abandoned for at least 50 years. We monitored the plots for four years, and collected data on species composition, photosynthetically active radiation (PAR) and red∶far-red ratio (R∶FR), soil moisture and compaction. We estimated community weighted means (CWM) for height, habit, life cycle, seed mass and SLA. Neither compaction nor soil moisture were modified by the treatments, while PAR and R∶FR increased in all treatments in comparison to the Control and Faeces treatments. The floristic composition of all treatments, except for Faeces, converged over time, but deviated from that of the Control. The functional traits displayed the trends expected in the presence of grazing: loss of erect species and increased cover of short species with light seeds, with rosettes and prostrate habit. However, contrary to the results in literature, SLA was lower in all the treatments than Control plots. Like the results for floristic composition, all treatments except for Faeces converged towards a similar functional composition at the end of the four year period. The results of this study show the initial evolution of a Mediterranean plant community in the presence of grazing, driven primarily by the destructive action of livestock. These actions seem to directly affect the rates of extinction/colonization, and indirectly affect the light environment but not the soil conditions. However, their effects on floristic and trait composition do not seem to differ, at least at the small spatio-temporal scale.

## Introduction

Livestock grazing influences vegetation through a series of direct and indirect effects on individual plants and on the environment, respectively, which have major consequences on the composition and structure of grassland communities at several hierarchical levels. For example, at the individual level, grazing produces changes in plant physiology and morphology (e.g. [1]), while at the population level, it can influence species colonization and extinction rates [2]. As a result, the effect of grazing is reflected in changes in the species composition and richness of the grassland communities [3]–[5], although these effects are often dependent on habitat productivity [6], [7]. Additionally, the effect of herbivores on species composition and richness can also depend on herbivore size [8]. For instance, Bakker et al. [8] found that assemblages including large herbivores increased plant diversity at high productivity levels but decreased diversity at low productivity levels; on the other hand, small herbivores did not have consistent effects along a productivity gradient ranging from 60 to 500 g/m^2^/year.

The influence of grazing on the species composition and richness of grasslands has been studied by means of grazing exclusion experiments [9], [10], comparisons between adjacent grazed and abandoned farms [5], [11] and gradients of grazing pressure [7], [12]. However, in the current context, in which the consequences of land use changes are increasingly concerning the scientific community, it is critical to improve our capacity to predict the effects that the different drivers of global change have on ecosystems. As a result, ecologists are increasingly using approaches based on the functional traits of organisms, i.e. measurable characteristics of species linked to their fitness and their effects on ecosystems, as a means to address some of the most fundamental and applied questions in ecology [13]. Functional traits approaches provide a more mechanistic point of view than the use of species identities alone, allowing the comparison between different ecosystems. Consequently, there is a growing interest in the study of plant trait responses to grazing [5], [12]–[15]. From these studies, we know that the effect of grazing on functional traits depends on the climate and the history of herbivory at the biogeographical scale, and only a few patterns have emerged at a global level. Among these patterns associated to grazing are the increase in the cover of shorter plants and of those with stolons or rosette forms, as well as the increase of annual species and the decrease of perennial species [16]. Nevertheless, fine scale factors such as topography and soil characteristics can shape community trait distribution values at local scales [17].

However, although grazing is considered to be a single factor in most studies that analyze the effects of grazing on vegetation (e.g. [7], [16]), livestock grazing encompasses different factors that have specific effects on vegetation and environmental features. These factors are primarily defoliation (herbage removal), faeces and urine deposition and trampling.

Defoliation, which is probably the most obvious component of livestock grazing, is a direct disturbance that consists on the loss or damage of photosynthetic tissue. Tissue loss leads to a reduction in the growth, reproductive performance and/or survival of the affected individuals [1], [18], [19]. In order to cope with defoliation, plants adopt two contrasting strategies: avoidance and tolerance [20]. Avoidance strategies seek to minimize the “frequency and/or intensity of herbivory by reducing plant palatability and accessibility” [20]. Plants that adopt this strategy are usually short, have small and unpalatable leaves or rosette growth forms [16]. In contrast, tolerance to herbivory is defined as the ability of plants to rapidly regrow after defoliation; tolerant plants therefore present functional traits associated with a high growth rate, such as a high specific leaf area (SLA). The prevailing strategy is context-dependent, with avoidance favoured over tolerance in conditions of low productivity (low water or nutrient availability) and vice-versa [16]. Defoliation is also associated with indirect effects because it modifies the light conditions in which plants germinate and grow, a feature that can have major implications for the species composition of plant communities [21], [22]. In addition, herbivore selectivity can alter species competition and dominance [23], [24].

Faeces and urine deposition by livestock plays an important role in grazed systems, because it increases soil fertility, which in turn can have major effects on the average functional trait values of plant communities and trigger significant changes in the abundances of plant species [25], [26]. High fertility favours species with traits associated with a rapid uptake of available resources, such as high SLA and high leaf nutrient concentrations [27]. Faeces deposition is also considered an important disturbance agent because it creates gaps that are subsequently colonized by the surrounding vegetation or by seeds present in the soil seed bank or in the dung pat itself [25], [28]. Several studies have underscored the importance of dung-dispersed seeds for the colonization of many species [28]–[32]. Faeces leachates also have different effects on the germination of different species, which can result in changes in the species composition of the affected communities [33].

Trampling produces physical damage to plants, reducing their cover and biomass [34], [35], ultimately leading to gap creation, greater soil compaction and soil density and reductions in pore size, especially in clay and wet soils [34], [36], [37]. Changes in soil characteristics result in a lower infiltration rate, associated with increased runoff and erosion [38]. Trampling also disrupts root growth and the production of new shoots of the affected plants [34], [39]. Some specific traits enable certain plants to cope better with trampling. For example, whereas trampling reduces the abundance of forbs [40], erect and woody plants, it increases the abundance of graminoids, and also favours short species over tall ones [41].

Very little is known about the effects of the various grazing factors and their interactions on vegetation. The results of previous studies that have isolated livestock components suggest that defoliation is the factor with the most important effects, whereas faeces addition seems to have little influence on species composition [42], [43]. However, these studies were performed in previously grazed grasslands [42], [43], which means that grazing had already filtered the pool of species available in these communities. To our knowledge, no study has experimentally tested the effects of the different grazing factors in grazing-abandoned systems, where the existing pool of species is un-influenced by land-use filters. The experimental application of different livestock factors in these communities should have a much greater effect than on communities subjected to grazing or in which grazing practices have recently been abandoned, helping to highlight the effects of the different grazing factors.

The present paper aims to separate the effects of the grazing factors (defoliation, faeces addition and trampling) on species richness and on the taxonomic and trait compositions of grazing-abandoned areas, mediated by environmental filters such as light availability, soil compaction and soil fertilization due to faeces deposition. In a five-year field experiment with a factorial design, these activities were simulated in abandoned grassland communities in the centre of the Iberian Peninsula. Specifically, we hypothesize that: (1) livestock factors associated with removal or damage of photosynthetic tissue, i.e. defoliation and trampling, increases the availability of light in the studied communities; (2) given that the study area has not experienced grazing in several years before the experiment started, all the studied factors have marked effects on the species composition of the communities, but the final composition of these communities should differ between the different grazing activities; (3) the different treatments select for functional traits adapted to cope with different conditions. We thus expect defoliation to select for short or annual species and for species with growth forms associated with grazing avoidance strategies, such as rosette or prostrate forms. We also expect that the increased productivity associated with faeces deposition selects for species that are adapted to rapid resource use, such as those with high specific leaf area. Finally, we expect trampling to select for graminoids and short species, and to reduce the abundance of woody species.

## Materials and Methods

### Ethics statement

The study was performed on public land with free access in the Pedrezuela municipality. No permission was required to enter or research in this area. This study did not involve or affect any endangered or protected species.

### Study area

The study area is 35 km north of Madrid, Spain (40°43′N and 3°39′W, ca. 900 m asl). The continental Mediterranean climate has a harsh summer drought, 550 mm of annual rainfall and 13°C mean annual temperature. The soils are shallow and poor (80% sand, 20% silt+clay [44]), developed over a gneiss substrate. The vegetation consists of camephyte shrubs (*Lavandula stoechas* subsp. *pedunculata*; to which we will refer as *Lavandula* onwards), and acidophilous grassland in open patches, where annual species abound. The grassland productivity is very low (between 49 and 342 g/m^2^/year) [45]. The study area has not been grazed by livestock for the last 50 years [46]. Previous studies did not show differences in species richness between adjacent grazed and ungrazed plots, but did show differences in species composition, with higher % of annual species in grazed than ungrazed plots [11], [44]. In the grazed areas, the average stocking rate is 0.4 LU/ha with continuous grazing all year round [30].

### Plots and treatments

A random block design was applied, using seven blocks and seven treatments to simulate the different types of livestock factors. A similar design has been used previously to simulate effects of cattle grazing [42], [43]. In autumn 2004, seven 15.25 m^2^ blocks were defined, 30 m to 787 m apart from each other, in an environmentally homogeneous, flat area. Seven 1.75×1 m^2^ plots were set 0.5 m apart to avoid edge effects. The following treatments were applied:

Defoliation (D): all vegetation in the plot was mown to a height of about 5 cm, similar to grazed farms adjacent to the study area with an average livestock density of 0.4 LU/ha.

Trampling (T): the ground was trampled using 40×30 cm^2^ boards strapped to the user's boots. In each treatment application, 1000 steps (571 steps/m^2^) were taken at a pressure of 0.054 kg/cm^2^ for each step. This treatment was similar to the one applied by Kohler et al. [42].

Faeces (F): faeces were collected each winter from grazed farms and, after oven drying at 50°C, they were pulverized and fed through a 500 µ sieve. Once a year, 250 g of this powdered dung was spread evenly in each plot. This amount is roughly equivalent to the material deposited each year by cattle in an equivalent area to the plot, at a 0.4 LU/ha stocking density [30].

Combination of Defoliation and Trampling (DT).

Combination of Defoliation and Faeces (DF).

Combination of Faeces and Trampling (FT).

Control (C).

**Figure 1:**
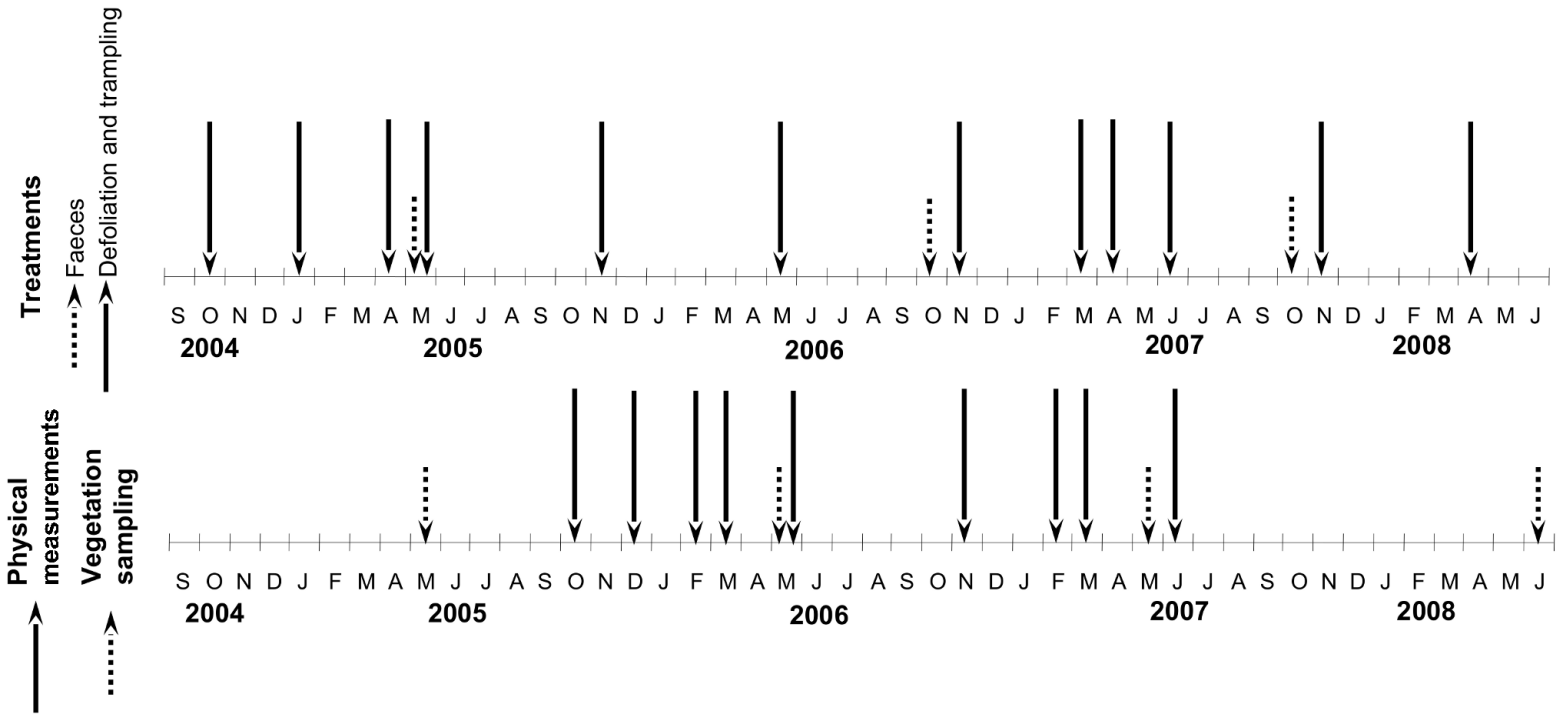
Treatments and sampling chronogram. Dates of the application of treatments (Faeces, Defoliation and Trampling), physical measurements (R∶FR: red∶far-red ratio; PAR: Photosynthetic active radiation; soil moisture; soil compaction) and vegetation sampling.

All treatments were applied on the dates specified in Fig. 1. The Defoliation and Trampling treatments were only carried out when the vegetation height permitted defoliation (over 5 cm), and always leaving at least one month for recovery between two consecutive applications.

### Floristic data

Floristic inventories on each plot were conducted each spring between 2005 and 2008 (Fig. 1). In order to minimize any possible border effect in the vegetation, we set three permanent 20×20 cm^2^ quadrats, which were placed 20 cm apart in a regular arrangement in the centre of each plot. Species cover was quantified with five levels: 0: Absence; 0.5: presence of a single individual (only for herbaceous species with little cover), 1: 1–25% cover, 2: 25–50% cover; 3: more than 50 % cover. Subsequently, each species was given the median value of each type of cover and averaged for each plot and year values of its three quadrats. Species richness was calculated per each plot and year as the number of unique species found on the three quadrats of each plot. Nomenclature follows [47].

### Functional traits

Reproductive and vegetative plant functional traits at the species level were measured in previous studies in the study area following protocols described by [48], or taken from local floras [49], to detect general trends in trait composition in relation to the simulated herbivores activities ([42], [43] Table 1). For each plot, we calculated the community weighted mean (CWM) values of each trait:
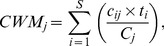
(1)where S is the number of species found in the plot, c_ij_ is the cover of species i in plot j, C_j_ = ∑c_ij_ and t_i_ is the average trait value for species i. CWM is generally accepted as an indicator of the average trait values of the dominant species in a community [13]. For qualitative traits (e.g. growth form), the CWM for each plot was expressed as the proportion of cover occupied by each of the trait categories.

**Table 1:** Plant functional traits.

Character	Variable	Units or possible values	Percentage of taxa with attribute	Source
Height	Quantitative	cm	85	[11]
SLA	Quantitative	mm^2^/mg	85	[11]
Seed weight	Quantitative	mg	100	[60]
Habit	Qualitative	Bulb, Graminoid, Erect, Rosette, Prostrate	100	[49]
Longevity	Qualitative	Annual, Perennial	100	[49]

### Physical measurements

In order to monitor the effect of the treatments on the physical environment of the plots, data on the light environment and soil physical properties were collected throughout the experiment (Fig. 1). Photosynthetically active radiation (PAR) was measured using a Quantum HD 9021 photoradiometer connected to a 5 cm high HD 9021 RAD/PAR probe (Delta OHM, Caselle di Selvazzano, Italy). The red∶far-red ratio (R∶FR) was measured with a 1 cm high fibre optic probe and a SKR 100/116 reader (Skye Instruments Ltd., Shropshire, UK). The soil water content was measured with a ThetaProbe ML2 sensor and a Theta HH1 Meter (Delta OHM, Caselle di Selvazzano, Italy).Soil compaction was estimated using an IB penetrometer (Eijkelkamp Agrisearch Equipment BV, Giesbeek, Netherlands).

Throughout the sampling periods, three light measurements were made in each floristic sampling quadrat, along with nine additional measurements in the open air, above the vegetation, in each plot. Soil water content and soil compaction were measured in ten points per plot on each occasion, always away from the sample quadrats, in order to avoid disturbance to the vegetation. However, soil water content measurements could not be performed in March and May 2006, because of the extreme hardness of the ground caused by long-lasting drought periods.

In order to estimate the effect of the Faeces treatment on the soil characteristics, at the end of the experiment, we collected soil samples in all the Faeces and Control plots using 5 cm diameter ×10 cm deep core samplers. Samples were air dried and sieved through 2 mm mesh. Following MAPA [50], we determined organic matter, total N, exchangeable P and K, sand and clay proportions and readily available water (amount of water between “field capacity” and “wilting point”).

### Statistical Analysis

Linear mixed effects models with temporal pseudoreplication [51] were used to analyse the effects of the treatments on the physical variables and on the log transformed species richness per plot. Treatment and measurement date, as well as the treatment by date interaction, were entered as fixed factors, and the nested effect of Block/Treatment as random factors, allowing the effect of date to vary between blocks. The lme function of the nlme package [52] in R version 2.15.1 [53] was employed. We also took into account possible autocorrelation structures between the measurements from different dates (corARMA or moving average autocorrelation, corAR1 or level 1 autocorrelation). For each response variable, we performed a model simplification, selecting the most parsimonious model according to the Akaike Information Criterion (AIC). The use of other criteria to select the best model (Bayesian Information Criterion and likelihood ratio tests) yielded the same results (Table S1 and Fig. S1). When significant effects of treatments were found, an analysis of multiple orthogonal comparisons was done to find homogeneous groups by means of the glht function of the multcomp package for R [54]. Soil features were analysed using paired t-tests. Each of the seven blocks was considered as a sampling unit, and the two measurements (Faeces and Control treatments) in each block were treated as repeated measures.

The floristic composition and CWM of the traits during the four years were analysed with Principal Response Curves (PRC). The PRC for floristic composition was performed on the matrix of 196 observations ×117 species, while for the PRC for functional features we used a matrix with 196 observations ×9 traits. The PRC were the result of a redundancy analysis (RDA), in which interaction between treatments and years were the explanatory variables and years the covariables [55]. The level of significance of the first axis of the RDA was established with a Monte Carlo permutations test with 499 permutations of the plots (n = 49) within the four years [55]. Species cover values were previously transformed to their natural logarithms and centered to avoid the effect of overestimation of the relative abundance of species with a high cover index, due to the unequal cover classes used in the sampling [55]; CWM values were transformed to their natural logarithms, centred and standardized for the above-mentioned reasons.

The significance of the second axis of the principle response curves was checked by including the scores of the plots as covariates in a second redundancy analysis using the same parameters as those employed in the analysis of the first axis [55]. Both PRC were performed using the *vegan* package for R [56].

## Results

### Physical Parameters

Among the physical variables, the treatments had no significant effect on soil moisture, which increased over time, or soil compaction, which decreased, as shown by the linear-mixed effects models (Table 2, 3 and 4). Additionally, the paired t-test did not reveal any significant effect of the Faeces treatment on the analysed soil parameters (Table 5).

**Table 2:** ANOVAs of linear mixed effects models. F and P values are indicated for each model. R∶FR: red∶far-red ratio; PAR: Photosynthetic active radiation. In the case of PAR2, the treatments were grouped as Disturbed (D, T and their combined treatments) and Undisturbed (Control and F). Species richness: log (species richness per plot).

		Treatment			Date
Variable	F	*P*		F	*P*
R∶FR	9.57	<0.0001			
PAR	6.63	0.0104		6.6338	0.0104
PAR2	52.20	4×10^−4^		50.4753	<0.0001
Soil compaction	0.38	0.8902		304.9933	<0.0001
Soil moisture	0.97	0.4621		189.8849	<0.0001
Species richness	4.68	0.0013			

**Table 3:** Parameter estimates for linear mixed effects models of the physical variables D = Defoliation, T = Trampling, F = Faeces. The intercept shows the estimation of the Control on the first measurement date (where this date is part of the minimal model). R∶FR: red∶far-red ratio; PAR: Photosynthetic active radiation; PAR2: PAR model with reclassification of the treatments as in Table 2; Date: Sampling year. Species richness: Log (species richness per plot).

	Value	Std. Error	df	t-value	p-value
**R∶FR**					
(Intercept)	0.6562	0.0276	391	23.7730	0.0000
D	0.1309	0.0275	36	4.7687	0.0000
DT	0.1918	0.0275	36	6.9852	0.0000
F	0.0451	0.0275	36	1.6428	0.1091
FD	0.0924	0.0275	36	3.3662	0.0018
FT	0.1107	0.0275	36	4.0311	0.0003
T	0.1346	0.0275	36	4.9029	0.0000
**PAR**					
(Intercept)	227.0651	51.7789	390	4.3853	0.0000
D	182.0164	40.5026	36	4.4939	0.0001
DT	188.9289	40.5026	36	4.6646	0.0000
F	22.4012	40.5026	36	0.5531	0.5836
FD	203.4610	40.5026	36	5.0234	0.0000
FT	173.7750	40.5026	36	4.2905	0.0001
T	209.3818	40.5026	36	5.1696	0.0000
Date	31.0391	12.0511	390	2.5756	0.0104
**PAR 2 Treatments**					
(Intercept)	238.0509	39.2006	425	6.0726	0.0000
Disturbed	180.5855	25.0285	6	7.2152	0.0004
Date	31.0274	4.3672	425	7.1046	0.0000
**Soil Moisture**					
(Intercept)	0.1267	0.0080	334	15.9037	0.0000
D	0.0049	0.0074	36	0.6677	0.5086
DF	0.0099	0.0074	36	1.3440	0.1874
DT	0.0122	0.0074	36	1.6549	0.1066
F	0.0081	0.0073	36	1.1133	0.2730
FT	0.0119	0.0073	36	1.6316	0.1115
T	0.0023	0.0072	36	0.3211	0.7500
Date	0.0111	0.0008	334	13.7799	0.0000
**Soil Compaction**					
(Intercept)	34.4451	1.1056	390	31.1544	0.0000
D	−0.7255	0.8193	36	−0.8856	0.3817
DF	0.2566	0.8193	36	0.3132	0.7559
DT	−0.3670	0.8373	36	−0.4383	0.6638
F	0.3668	0.8193	36	0.4477	0.6571
FT	0.1951	0.8193	36	0.2382	0.8131
T	−0.0451	0.8193	36	−0.0551	0.9564
Date	−1.9321	0.1093	390	−17.6745	0.0000
**Species Richness**					
(Intercept)	2.9047	0.05265	147	55.1684	0.0000
D	0.1607	0.06543	36	2.4650	0.0190
DF	0.07857	0.065435	36	1.2009	0.2376
DT	0.0786	0.0654	36	−1.2551	0.2175
F	0.02441	0.0654	36	0.4189	0.6777
FT	−0.070496	0.0654	36	−1.0761	0.2890
T	−0.1251	0.0654	36	−1.9124	0.0638

**Table 4:** Variances of fixed and random factors and residuals of linear mixed effects models. Percentage of total variance explained by each group of factors in brackets. R∶FR: red∶far-red ratio; PAR: Photosynthetic active radiation.PAR2: PAR model with reclassification of the treatments as in Table 2. Species richness: log (species richness per plot).

Variable	Random	Fixed	Residual
R∶FR	2.99×10^−3^ (5.1)	3.39×10^−3^ (5.8)	52.03×10^−3^
PAR	10841.71 (14.4)	13248.72 (17.6)	51255.45
PAR2	4290.53 (5.8)	13103.04 (17.8)	56054.33
Soil compaction	4.7869 (3.0)	24.9773 (15.1)	135.7551
Soil moisture	1.69×10^−4^ (2.4)	6.65×10^−4^ (9.6)	60.76×10^−4^
Species richness	1.44×10^−2^ (12.8)	8.63×10^−3^ (7.7)	8.91×10^−2^

**Table 5:** Mean and standard deviation of soil features in the Faeces and Control treatments. t-Statistics and P-value of respective paired t-tests are also shown. N = 7 for all cases.

Variables	Faeces (mean±SD)	Control (mean±SD)	t	p
Organic Matter (%)	1.77±0.45	1.76±0.40	0.085	0.935
Total N (%)	0.17±0.09	0.20±0.04	−0.84	0.432
Exchangeable P (ppm)	7.86±1.77	6.71±1.25	−1.22	0.268
Exchangeable K (ppm)	159.86±43.78	151.14±35.01	−0.56	0.597
Sand (%)	78.03±3.76	75.74±1.82	−2.38	0.055
Clay (%)	6.17±1.44	5.96±1.18	−0.35	0.738
Readily Available Water (%)	6.42±1.63	5.72±0.85	−2.03	0.089
pH	6.16±0.18	6.06±0.16	−1.45	0.197

**Figure 2:**
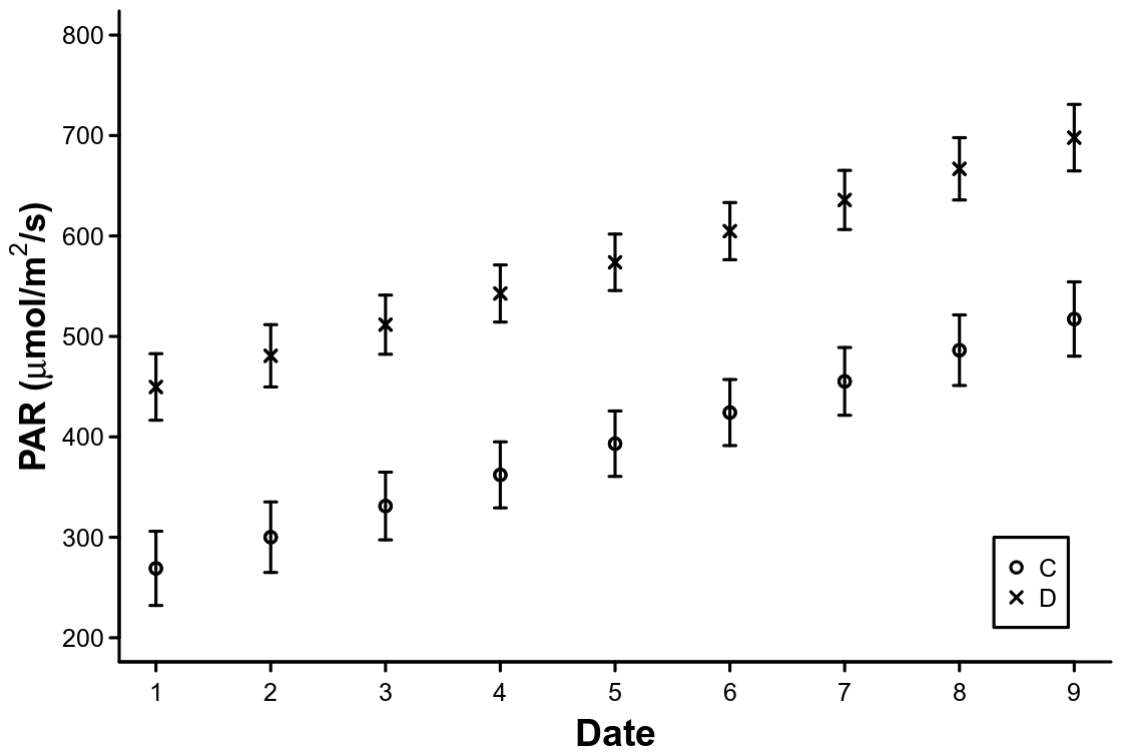
Estimates of Photosynthetic active radiation (PAR) on each measurement date for the two treatment groups. Bars are ± standard error. Treatment groups: Undisturbed (C - Control and Faeces), Disturbed (D – rest of treatments).

The PAR increased significantly in all treatments compared with the Control, except for Faeces. However, post-hoc tests revealed no differences between treatments. We therefore decided to define two treatments, one with Control and Faeces (undisturbed) and another with the rest (disturbed) (Table 2, 3 and 4; PAR2 treatment). In this case, PAR was significantly higher in the disturbed plots than in the undisturbed ones (Fig. 2).

**Figure 3:**
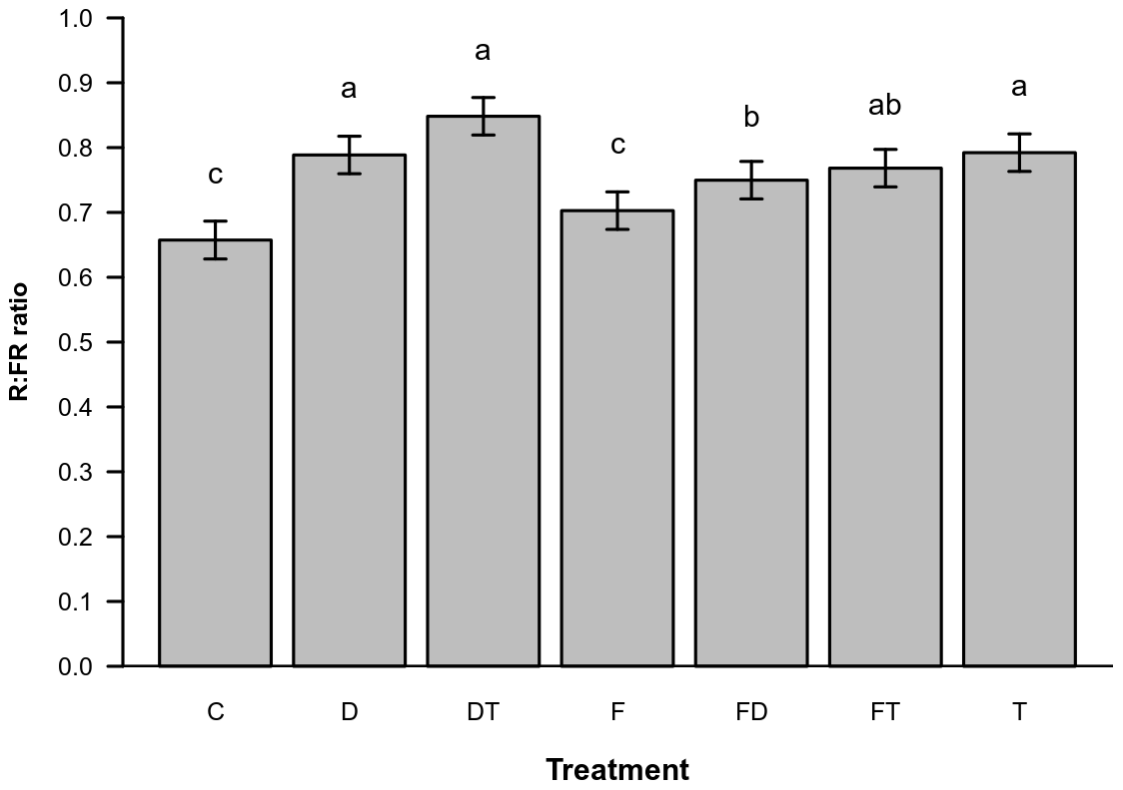
Estimated red∶far-red ratio (R∶FR) for each treatment and standard errors. Letters above bars indicate homogeneous groups (<0.05).

The R∶FR showed significant effects of the treatments (Table 2, 3 and 4). The subsequent multiple orthogonal comparisons showed that R∶FR increased in all treatments except for Faeces. Defoliation, Trampling and their combination resulted in the greatest increase in R∶FR, whereas the combination of Faeces and Defoliation resulted in a slight increase compared with the Control. The combination of Trampling and Faeces caused an intermediate increase in the R∶FR values (Fig. 3).

### Species richness and composition

The only treatment that caused a change in the average species richness per plot compared with the Control was Defoliation, with an increase of 1.2±1.1 species (mean ± SE; exponential transformed from Table 2, 3 and 4).

**Figure 4:**
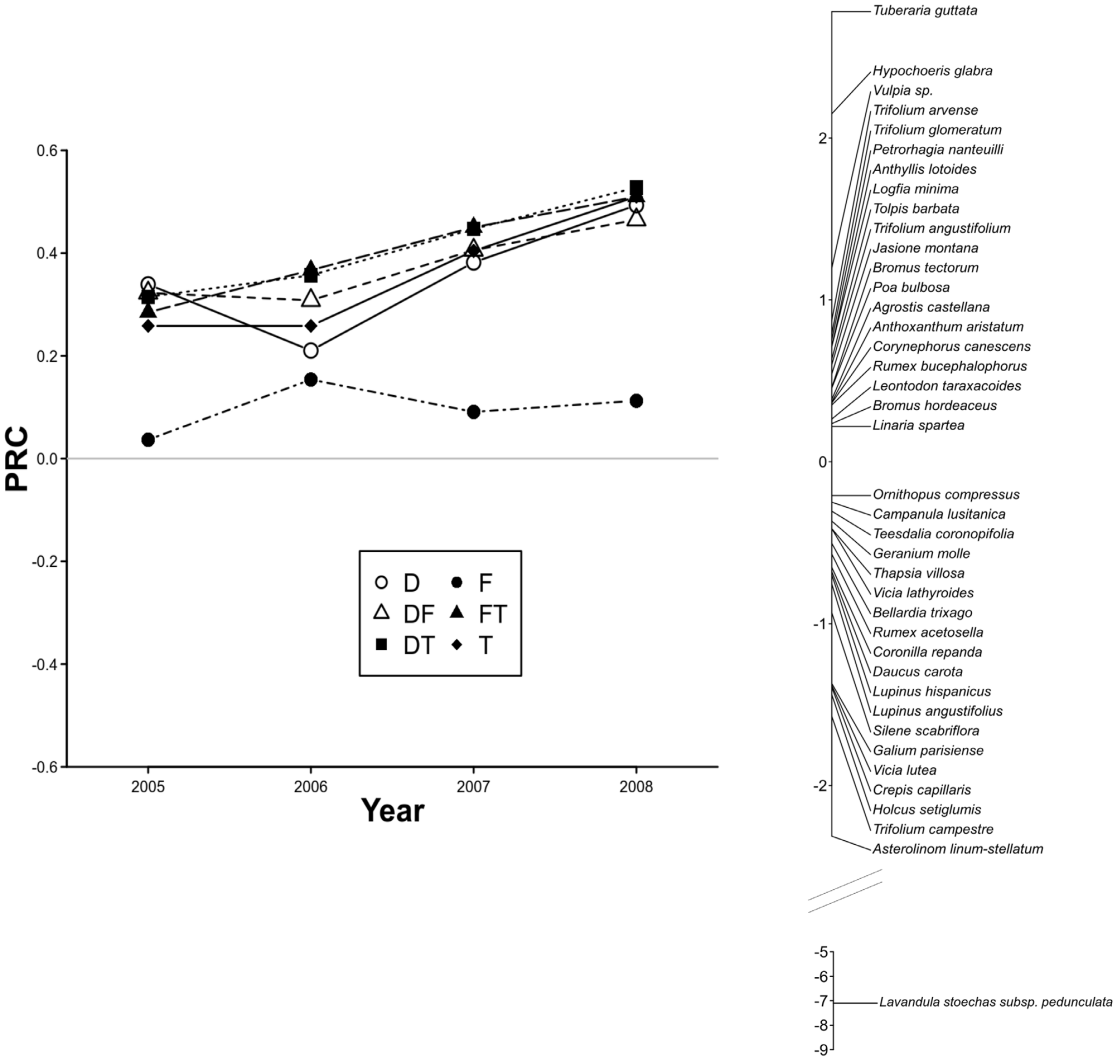
First axis of the principal response curves for change in plant species' cover. Results in plots subjected to Defoliation (D), Trampling (T), Faeces (F) and their combined treatments (DF, DT and FT) between 2005 and 2008, compared to the Control plots (C, baseline 0). Species weight shown on the right of the diagram represent affinity of each species with the response shown. For clarity, only species with scores higher than 0.2 and lower than −0.2 are plotted.

The Monte Carlo test for the first axis of the principal response curves analysis (PRC) was significant (F = 13.39, p = 0.005), explaining 5.6% of the variability in the data. The second axis was not significant (F = 3.13, P = 0.870). The floristic composition of the Defoliation, Trampling and their combined treatment diverged from the Control plots in the course of the four year sampling period, while the Faeces treatment scarcely differed from the Control in the same period (Fig. 4).

*Lavandula* made the greatest contribution to this differentiation (Fig. 4). In the final year, 2008, the cover of this species for all treatments, except for Faeces, was 97% lower than in the Control. In the same year, the cover of *Asterolinon linum-stellatum* was 69% lower than the Control in all treatments except for Faeces (Fig. 4).

*Tuberaria guttata*, *Hypochoeris glabra* and various species of the *Vulpia* genus stood out amongst the species with a larger percentage of cover in all treatments, except for Faeces, compared with the Control, with average increases of 4.0%, 2.9% and 1.8%, respectively, at the end of the experiment (Fig. 4).

### Functional traits

**Figure 5:**
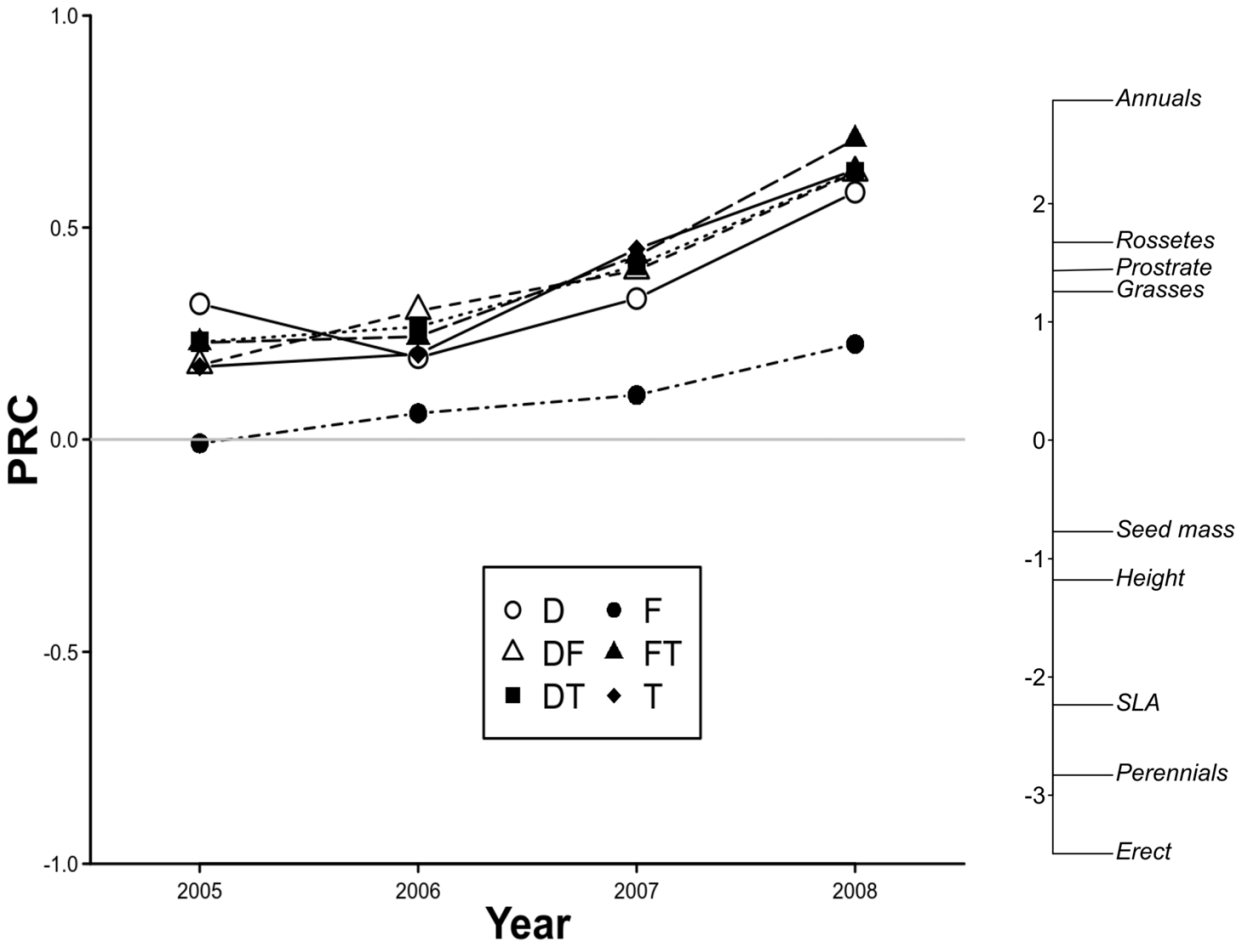
First axis of the principal response curves for change in functional traits. Results in plots subjected to Defoliation (D), Trampling (T), Faeces (F) and their combined treatments (DF, DT and FT) between 2005 and 2008, compared to the Control plots (C, baseline 0). Three of the traits are continuous variables (height, seed mass, SLA), while the other two (habit/life form, life cycle) are characterized by the weighted averages of percentage cover for the different categories (Table 1).

The first PRC axis was significant according to the Monte Carlo permutations test (F = 2.21, p = 0.005), explaining 13.07% of the data variability. The second PRC axis was not significant (F = 0.76, p = 0.92). All treatments related to grazing gradually increased their divergence in functional composition compared with the Control plots during the experimental period (Fig. 5). The plots under simulated grazing factors presented a greater cover of short species with light seeds, lower SLA values, and a greater cover of annuals, prostrates, rosettes and grasses compared with the Control plots (Fig. 5 and Table 6). The Faeces treatment differed less from the Control than the others (Fig. 5), although this differentiation was more obvious than in the case of floristic composition.

**Table 6:** Means and standard errors of functional traits and attributes for each treatment (C = Control, D = Defoliation, T = Trampling, F = Faeces) in 2008.

	Treatment						
	C	D	DF	DT	F	FT	T
**Height (cm)**	13.04±1.56	11.44±1.10	11.43±1.78	10.03±0.24	14.80±2.81	10.99±1.06	9.45±0.96
**SLA (mm^2^/mg)**	27.84±1.07	23.50±0.70	23.55±0.40	23.44±0.43	26.85±1.15	23.03±0.67	23.20±0.91
**Seed mass (mg)**	1.02±0.33	0.76±0.15	0.90±0.13	1.30±0.59	1.30±0.26	1.08±0.35	1.11±0.58
**Habit/Life form**							
Grasses	0.10±0.04	0.10±0.04	0.16±0.05	0.12±0.04	0.18±0.06	0.21±0.05	0.24±0.07
Erect	0.67±0.03	0.44±0.04	0.39±0.03	0.36±0.03	0.50±0.06	0.31±0.05	0.31±0.02
Rosettes	0.15±0.03	0.30±0.04	0.28±0.04	0.31±0.04	0.20±0.02	0.31±0.04	0.27±0.04
Prostrate	0.09±0.03	0.16±0.03	0.17±0.04	0.21±0.04	0.12±0.03	0.17±0.02	0.18±0.05
**Life cycle**							
Annuals	0.55±0.08	0.88±0.02	0.89±0.04	0.84±0.04	0.64±0.07	0.88±0.05	0.79±0.08
Perennials	0.45±0.08	0.12±0.02	0.11±0.04	0.16±0.04	0.36±0.07	0.12±0.05	0.21±0.08

## Discussion

As expected, the treatments that removed plant biomass, Defoliation and Trampling, increased the amount of photosynthetic active radiation (PAR) at the ground level. The Faeces treatment and the Control consistently showed lower values of this parameter than the other treatments. Similarly, Defoliation and Trampling increased the R∶FR values above those of the Control and the Faeces treatment. These changes in light conditions might explain the effects of these treatments on the floristic composition. Previous experimental studies in the same study area have shown that the germination response of species classified as *grazing increasers* (based on a higher relative abundance in the presence vs absence of grazing) is differently affected by changes in light quality (R∶FR) but not in light quantity (PAR) than that of *grazing decreasers*. In this previous study, *increasers* had lower germination percentages at low R∶FR ratios, possibly as a mechanism to avoid germination in highly competitive environments. However, there were no differences in the response to changes in PAR between the two grazing response groups: germination was inhibited in both groups at higher light intensities [21].

The treatments did not affect the physical characteristics of the soil (compaction and moisture content). Since it has been found that livestock trampling causes soil compaction and reduces infiltration [34], [37], our results suggest that the level used in our study was below the necessary intensity. Other methods to simulate trampling, that apply much higher pressures, such as the ones used by Di et al. [57] or Dunne et al. [58] are probably better alternatives to achieve levels of soil compaction similar to those actually caused by livestock action. Nevertheless, although it did not affect soils compaction, our Trampling treatment had an important effect on vegetative tissue, which is one of the most important consequences of trampling (e.g. [58]).

The only treatment with a significant effect on species richness per plot was Defoliation (D). The almost complete disappearance of *Lavandula*, the only woody species on these plots, together with the significant increase in the availability of light at 5 cm above ground level, suggest that defoliation primarily affects dominant species, and that an increase in the availability of resources such as light reduces the competitive pressures faced by subordinate species. This result is in agreement with those reported by Bonanomi et al. [59], who found increased diversity in Mediterranean grasslands linked to defoliation, possibly related to the decrease in the dominance of perennial grasses and the presence of woody species; however, it is important to note that higher grazing pressures than those simulated in our study can result in a reduction in species diversity, especially in low productivity conditions [7], [60]. However, other treatments such as Trampling or the combination of Defoliation and Trampling, which also caused an increase in the availability of light and a reduction of the dominant species, did not result in a similar increase in species richness. One explanation for this contradiction is that, while trampling increases the amount of light available to subordinate species, this effect is counteracted by increases in plant mortality, which could result in the lack of differences in species richness between grazed and ungrazed areas observed in previous studies in the same area [5], [44].

In contrast to previous studies in the same area, which found that the application of faeces in experimental plots produced an increase in species richness [61], our results showed no effect of the addition of excrement in this parameter, and a very marginal change in the floristic composition. This difference may be due to the use of spring faeces in the experiment by Traba et al. [61], with a high content of viable seeds. In contrast, we used winter faeces in the Faeces treatment in the present study, because we specifically intended to test the effect of soil fertilization by the addition of faeces, independently of the input of seeds transported by endozoochory. The combined results of these two studies suggest that the effect of faeces deposition on species richness at a very fine scale could be more probably related to endozoochorous dispersal than to fertilization, at least for the livestock densities (0.4 LU/ha) simulated in our experiment. Nevertheless, this conclusion should be considered with caution because, although it is known that grazing has a significant fertilization effect on the study area [5], [44], we did not find any fertilization effect after four years of Faeces treatment (Table 5). It is possible that this factor has a longer-term effect than the time scale used in the present study (four years). Furthermore, we acknowledge that the Faeces treatment in our experiment could differ from the actual action of cattle under field conditions. For instance, we used dried faeces, but it is important to consider that the bioavailability of nutrients might differ between fresh and dry faeces. Some authors have found that the availability of P in dung decreases with drying [62]. Nevertheless, our results are in good agreement with those of Kohler et al. [42], who applied a different fertilization treatment (manuring; containing fresh dung and urine), and found that its impact was much smaller than those of defoliation or trampling. In addition, since our study took place in a long-term grazing abandoned area, the lack of propagules from species adapted to take advantage of the new conditions may also underlie the lack of effects of the Faeces treatment. Finally, part of the effects of the faeces may be due to factors that were not taken into account in our experimental design, such as the lower grazing activity around the dung pats [26], or the colonization processes that take place after the decomposition of the Faeces [25], [28].

Composition in all treatments with the exception of Faeces converged over time, while their differences from the Control increased. These differences from the Control were primarily caused by the reduction in the cover of *Lavandula*, which was almost completely removed when the Defoliation and Trampling treatments were applied. *Lavandula* is a woody species that is known to be the main colonizing species after grazing abandonment in this study area [11]. Previous studies have shown that livestock eat flowers and new shoots of *Lavandula*, and that viable seeds of this species are present in sheep and cattle faeces [63], [64]. Our results confirm that this species is sensitive to defoliation and trampling and that the reintroduction of grazing in areas with a high cover of *Lavandula* probably results in a fast decline of its dominance.

The response of the different species has provided experimental support for the classification of some species as grazing increasers and decreasers in the study area [11], [44]. Besides *Lavandula*, the cover of the decreasers *Asterolinon linum-stellatum*, *Holcus setiglumis*, *Silene scabriflora*, *Coronilla repanda* and *Campanula lusitanica*, diminished in the Defoliation and Trampling treatments. On the other hand, the cover of the increasers *Hypochoeris glabra* and several *Vulpia* spp., increased in plots where simulated grazing disturbance treatments (D, F, and their combined treatments) were applied. However, there were also inconsistencies in the response of several species such as *Rumex acetosella*, classified as an increaser although its cover declined after the treatments, whereas the decreasers *Corynephorus canescens* and *Jasione montana*, were more abundant in the treatments than in the Control.

The application of the treatments had an effect on the functional composition of the community. All treatments favoured species with small seed weights, although this effect was lower in the Faeces treatment. Several authors have found an increase in small-seeded species during grazing in Mediterranean environments [10], [59], [65], and relate it with the adaptive advantage of small-seeded species for the colonization of gaps created by livestock, because of the seed mass-seed number trade-off (see also [43]). The increased average weight of seeds in the Control treatment is consistent with the detected reduction in the amount of available light in these quadrats, which probably favours large-seeded species, whose seedlings can survive better under competition for light [66].

Height was also lower in all the treatments, except the Faeces treatment, compared with the Control (Table 6). Several authors have observed a decrease in plant height with grazing both in Mediterranean environments [5], [11], [67]–[69] and at the global scale [16]. Simulated grazing increased the proportion of annuals, rosettes, and prostrate species, and reduced the cover of species with erect forms. However, it remains unclear whether this effect is caused by the removal of erect species in the Defoliation treatment [70] or by the greater importance of competition for light in the Control plots compared with the treatments [71].

Finally, the Control plots presented higher average values of SLA than the treatments plots, contrary to the general expectation of increased SLA under grazing conditions [15], [16], [72], but consistent with previous observations in the same area, where the positive effect of grazing was only significant for intermediate SLA values [5], [11]. Differences in SLA can be the result of trade-offs between different functions of the leaf such as photosynthesis, competition, storage, damage prevention and support, and the result of these trade-offs can vary with the environment [73]. Altogether, these results suggest that species with grazing-avoidance strategies are favoured by these types of treatments, which is consistent with the expected effect of grazing in dry and poor soil environments such as the study area [16].

### Concluding remarks

This study shows that various grazing activities have different effects on the environmental features, species richness, and taxonomical and functional compositions of the studied grasslands. Although other authors have previously isolated the effects of the different livestock factors on vegetation [42], [43], there are nevertheless major differences between those studies and the present experiment. First, Kohler et al. [42], [43] performed their studies in grazed areas, which implies that the species pool was already filtered by livestock factors. In contrast, our study was in grazing abandoned areas, and we therefore expected our simulated grazing activities to have a much greater effect by excluding species that cannot cope with these disturbances. We are aware of the possible effect of past grazing activities in the study area, which might influence the current species pool of the studied sites and, consequently, our findings. Nevertheless, although past land-uses have been found to influence diversity more strongly than current uses in some instances (e.g. [74]), previous research in the study area has shown that there are great floristic differences among grazed areas and areas that have not been grazed for a period similar to that of the area in which our experiment was performed [11], [44]. This indicates that the studied plant communities experience important changes after grazing abandonment in a relatively short period, which minimizes the possible impacts of past land-uses in our results.

We found that the Faeces treatment resulted in communities that were quite similar to those observed in the Control plots. Faeces deposition and decomposition is known to determine the formation of patches of some species [25], [26], and given that dung leachates have different effects on the germination of species with different grazing responses [33], we expected a greater effect of this treatment. The lack of propagules of grazing-increaser species, capable of take advantage of the conditions generated in the Faeces treatment might have been behind this negative result. Field experiments that include the addition of seeds of grazing-increaser species would be helpful to clarify this point.

In contrast, our results suggest that livestock activities that cause a loss of foliar tissue have a greater impact on species and functional trait composition than the increase in nutrient availability associated with faeces deposition. The treatments that included Defoliation or Trampling presented marked differences with the Control treatment, but were quite similar to each other. The clear effect of both activities contrasts with previous studies, which found no consistent effects of defoliation or trampling: while Kohler et al. [42] found that defoliation caused a much greater effect on species composition than trampling, the opposite result was found in the study of gap colonization [43]. These results show the importance of performing this kind of studies in grazing-abandoned areas where the livestock-imposed filter has not been previously applied. However, in areas subjected to grazing for a long time, there could be a divergence in the species and traits adapted to these two activities, which would explain the contrasting effects found by Kohler et al. [42], [43]. The convergence in species composition of the Trampling and Defoliation treatments over time found in our study suggests that species that can cope with defoliation are the same than those that can resist trampling. A similar result was found regarding functional trait composition, indicating a convergence in the trait values associated with resistance to defoliation and trampling. In this context, it is important to note that, unlike trampling, defoliation is a selective disturbance [23], [24], [75], and that the Defoliation treatment that we applied is non-selective. This feature, along with the failure of our Trampling to produce a significant soil compaction, can be behind the similar results yielded by our Defoliation and Trampling treatments.

## Supporting Information

Figure S1 Autocorrelation figures of the most parsimonious linear mixed models for red-far red ratio (RFR), soil compaction, soil moisture and log of species richness. Parameters for autocorrelation-moving average correlation structure (p and q), and the estimated autoregressive and moving average parameters (φ and θ) are also given.
(DOC)

Table S1 Selected mixed linear models of each physical parameter and log of species richness. The structure of the fixed effects is specified for each model (* interaction taken into account, + interaction not taken into account), the presence of a random factor (Date) and autocorrelation structure between measures on different dates (corARMA-moving average autocorrelation, order 1 corAR1-autocorrelation). Nesting of treatments in plots was considered in all models. Not all possible combinations are shown, either due to assumption of overparametrizations in models, or because autocorrelations were discarded after inspecting the autocorrelation diagrams. Selected minimum models shown in bold. AIC: Akaike Information Criterion; BIC: Bayesian Information Criterion; logLik: log Likelihood; L-ratio and p-value: results of ANOVAs of comparison between models.
(DOC)
