# Supplementary figures and images for: Dissecting the Effects of Simulated Cattle Activity on Floristic Composition and Functional Traits in Mediterranean Grasslands
Source: PLoS One. 2013 Nov 20;8(11):e79822. doi: 10.1371/journal.pone.0079822 (PMC3835893; doi:10.1371/journal.pone.0079822)

**Figure S1**

**
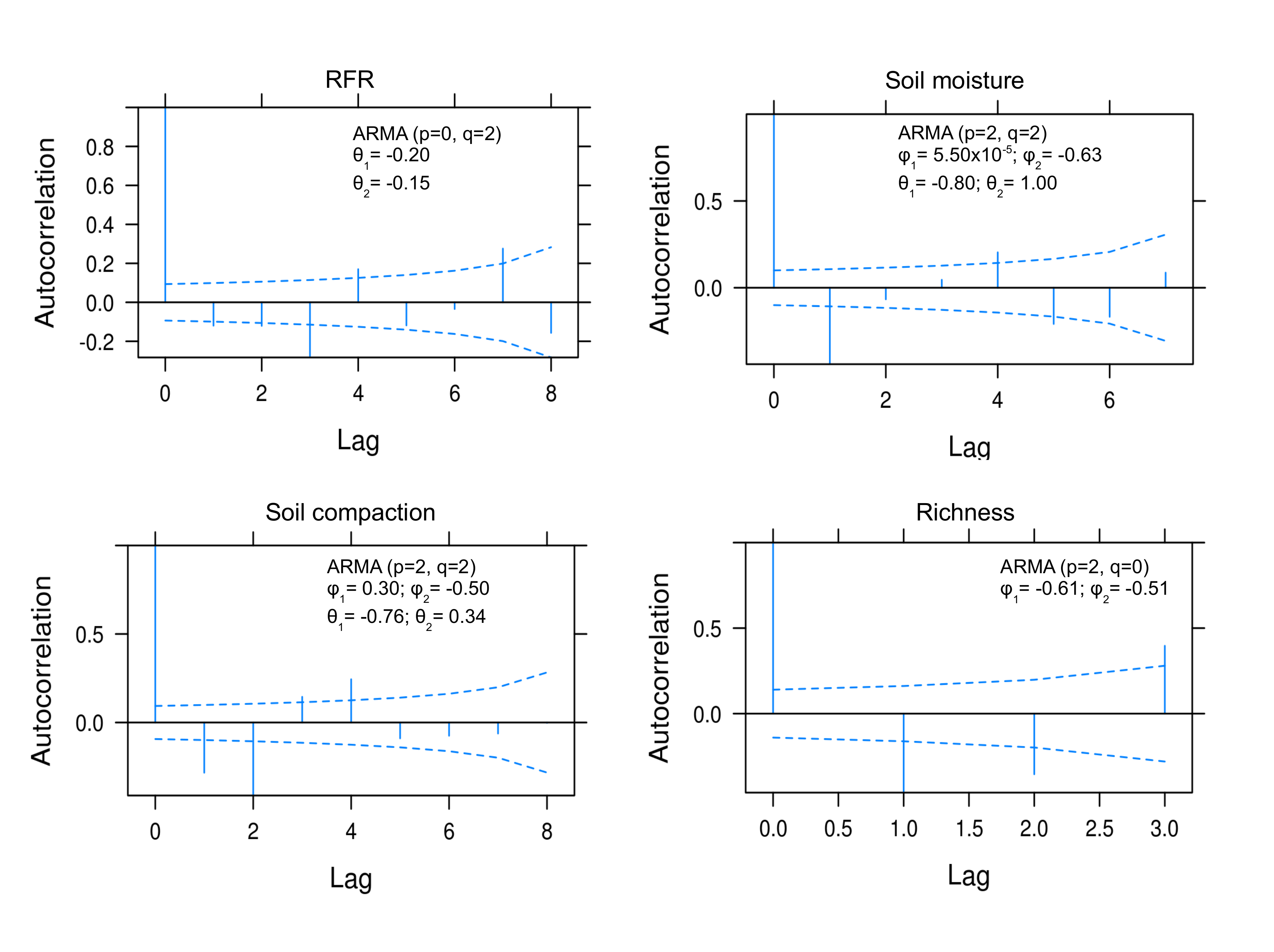
**

Supplement: Figure S1 — Autocorrelation figures of the most parsimonious linear mixed models for red-far red ratio (RFR), soil compaction, soil moisture and log of species richness. Parameters for autocorrelation-moving average correlation structure (p and q), and the estimated autoregressive and moving average parameters (φ and θ) are also given. (DOC) [file pone.0079822.s001.doc]
