# Supplementary material for: Dissecting the Effects of Simulated Cattle Activity on Floristic Composition and Functional Traits in Mediterranean Grasslands
Source: PLoS One. 2013 Nov 20;8(11):e79822. doi: 10.1371/journal.pone.0079822 (PMC3835893; doi:10.1371/journal.pone.0079822)
Supplement: Table S1 — Selected mixed linear models of each physical parameter and log of species richness. The structure of the fixed effects is specified for each model (* interaction taken into account, + interaction not taken into account), the presence of a random factor (Date) and autocorrelation structure between measures on different dates (corARMA-moving average autocorrelation, order 1 corAR1-autocorrelation). Nesting of treatments in plots was considered in all models. Not all possible combinations are shown, either due to assumption of overparametrizations in models, or because autocorrelations were discarded after inspecting the autocorrelation diagrams. Selected minimum models shown in bold. AIC: Akaike Information Criterion; BIC: Bayesian Information Criterion; logLik: log Likelihood; L-ratio and p-value: results of ANOVAs of comparison between models. (DOC) [file pone.0079822.s002.doc]

**Table S1**.

| **N** | **Fixed** | **Random** | **Correlation structure** | **df** | **AIC** | **BIC** | **logLik** | | | **test** | **L-ratio** | | **p-value** | |
| --- | --- | --- | --- | --- | --- | --- | --- | --- | --- | --- | --- | --- | --- | --- |
|  | **RFR** |  |  |  |  |  |  | | |  |  | |  | |
| 1 | Treatment*Date | Date |  | 21 | -18.06 | 67.76 | 30.03 | | |  |  | |  | |
| 2 | Treatment*Date | 1 |  | 17 | -26.06 | 43.41 | 30.03 | | | 1vs2 | 0.0000 | | 1 | |
| 3 | Treatment+Date | Date |  | 15 | -25.87 | 35.43 | 27.93 | | | 1vs3 | 4.1929 | | 0.6506 | |
| 4 | Treatment+Date | Date | corARMA(q=2) | 17 | -32.96 | 36.51 | 33.48 | | |  |  | |  | |
| 5 | Treatment+Date | Date | corAR1 | 16 | -26.52 | 38.87 | 29.26 | | | 4vs5 | 8.4401 | | 0.0037 | |
| 6 | Treatment | Date | corARMA(q=2) | 16 | -32.86 | 32.53 | 32.43 | | | 4vs6 | 2.1051 | | 0.1468 | |
| **7** | **Treatment** | **1** | **corARMA(q=2)** | **12** | **-40.86** | **8.19** | **32.43** | | | **6vs7** | **0.0000** | | **1** | |
|  | **PAR** |  |  |  |  |  |  | | |  |  | |  | |
| 1 | Treatment*Date | Date |  | 21 | 6071.76 | 6157.59 | -3014.88 | | |  |  | |  | |
| **2** | **Treatment+Date** | **Date** |  | **15** | **6064.53** | **6125.83** | **-3017.26** | | | **1vs2** | **4.7643** | | **0.5744** | |
| 3 | Treatment+Date | 1 |  | 11 | 6089.61 | 6134.56 | -3033.80 | | | 2vs3 | 33.0785 | | <.0001 | |
| 4 | Treatment | Date |  | 14 | 6067.74 | 6124.95 | -3019.87 | | | 3vs4 | 27.8661 | | <.0001 | |
| 5 | Treatment+Date | Date | corARMA(p=1,q=2) | 18 | 6064.12 | 6137.68 | -3014.06 | | |  |  | |  | |
| 6 | Treatment+Date | Date | corARMA(q=2) | 17 | 6063.78 | 6133.26 | -3014.89 | | | 5vs6 | 1.6644 | | 0.1970 | |
| 7 | Treatment+Date | Date | corAR1 | 16 | 6065.78 | 6131.17 | -3016.89 | | | 6vs7 | 3.9936 | | 0.0457 | |
|  | **PAR 2 Treatments** |  |  |  |  |  |  | | |  |  | |  | |
|  | Treatment+Date | 1 |  |  | 6059.47 | 6083.95 | -3023.74 | | |  |  | |  | |
|  | **Humidity** |  |  |  |  |  |  | | |  |  | |  | |
| 1 | Treatment*Date | 1 |  |  | -839.79 | -772.63 | 436.90 | | |  |  | |  | |
| 2 | Treatment+Date | 1 |  | 11 | -851.47 | -808.02 | 436.74 | | |  |  | |  | |
| 3 | Treatment | 1 |  | 10 | -841.44 | -801.93 | 430.72 | | | 2vs3 | 12.0389 | | 0.0005 | |
| **4** | **Treatment+Date** | **1** | **corARMA(p=2,q=2)** | **15** | **-992.21** | **-932.95** | **511.10** | | |  |  | |  | |
| 5 | Treatment+Date | 1 | corARMA(q=2) | 13 | -976.82 | -925.46 | 501.41 | | | 4vs5 | 19.3872 | | 0.0001 | |
| 6 | Treatment+Date | 1 | corAR1 | 12 | -921.88 | -874.48 | 472.94 | | | 5vs6 | 56.9360 | | <0.0001 | |
|  | **Compression** |  |  |  |  |  |  | | |  |  | |  | |
| 1 | Treatment*Date | 1 |  |  | 3462.02 | 3531.49 | -1714.01 | | |  |  | |  | |
| **2** | **Treatment+Date** | **1** | **corARMA(p=2,q=2)** | **15** | **3326.99** | **3388.30** | **-1648.50** | | |  |  | |  | |
| 3 | Treatment+Date | 1 | corARMA(p=1,q=2) | 14 | 3339.53 | 3396.75 | -1655.77 | | | 2vs3 | 14.5383 | | 0.0001 | |
| 4 | Treatment+Date | 1 | corARMA(q=2) | 13 | 3343.03 | 3396.16 | -1658.51 | | | 3vs4 | 5.4956 | | 0.0191 | |
| 5 | Treatment+Date | 1 | corAR1 | 12 | 3427.15 | 3476.19 | -1701.57 | | | 4vs5 | 86.1197 | | <.0001 | |
| 6 | Treatment | 1 | corARMA(p=2,q=2) | 14 | 3494.76 | 3551.98 | -1733.38 | | | 2vs6 | 169.7689 | | <.0001 | |
|  | **Species richness** |  |  |  |  |  |  | | |  |  | |  | |
| 1 | Treatment*Date | Date |  | 21 | 136.40 | 205.24 | -47.20 | | |  |  | |  | |
| 2 | Treatment+Date | Date |  | 15 | 131.73 | 180.90 -50.86 | | 1vs2 | 7.3297 | | | 0.2914 | |  |
| 3 | Treatment+Date | 1 |  | 11 | 123.73 | 159.79 | -50.86 | | | 2vs3 | 1.94x10^-8^ | | 1 | |
| 4 | Treatment | 1 |  | 10 | 122.94 | 155.72 | -51.47 | | | 3vs4 | 1.2135 | | 0.2706 | |
| 5 | Treatment | 1 | corARMA(p=2, q=2) | 14 | 108.89 | 154.79 | -40.45 | | | 4vs5 | 22.0486 | | 0.0002 | |
| 6 | Treatment | 1 | corARMA (p=2, q=1) | 13 | 106.89 | 149.51 | -40.45 | | | 5vs6 | 1.87x10^-10^ | | 1 | |
| **7** | **Treatment** | **1** | **corARMA (p=2, q=0)** | **12** | **104.89** | **144.23** | **-40.455** | | | **6vs7** | **1.49x10^-10^** | | **1** | |
| 8 | Treatment | 1 | corAR1 | 11 | 115.20 | 151.26 | -46.60 | | | 7vs8 | 12.3119 | | 0.0005 | |
